# Explanation Respecting Mr. Adams

**Published:** 1814-05

**Authors:** John Barrow

**Affiliations:** Admiralty


*For the Medical and Physical Journal.*

*Explanation respecting Mr. Adams; by* Mr. Barrow.

IN the last Number of the Medical and Physical Journal, I observe, in a paper under a concealed name, some remarks on a paragraph which appeared in the Courier Newspaper, relating to a successful operation for cataract, performed upon Mr. Turnbull, by Mr. Adams the oculist. As the author of those "remarks" expresses himself in rather ambiguous terms, and as I was the mean by which Mr. Adams's name was affixed to the paragraph in question, I deem it proper to make you acquainted with the following circumstance, to obviate any obloquy which might be supposed to attach to him by those who may consider it improper for a professional man's name to appear in a public newspaper.

Having witnessed the extraordinary success of Mr. Adams's new operations on several of the Greenwich pensioners, who appeared at this office, I was induced to recommend my friend Mr. Turnbull, whose sight was rapidly impairing, to consult Mr. Adams. He lost no time in doing so, and submitted to the new practice on one eye, the sight of which was totally gone. In the short space of three days he recovered the sight of that eye, which was considered by a near relative of his so extraordinary, that he brought me the paragraph in question, requesting me to give it to the editor of one of the daily papers. The name of Mr. Adams was not affixed to the paragraph: I therefore added it of my own accord, under the impression that it might be the means of others deriving similar benefit to that which Mr. Turnbull had received. I gave it to the editor of the *Sun*, and to no other, from which paper I apprehend it was copied into the *Courier*.

JOHN BARROW.

